# PyPNS: Multiscale Simulation of a Peripheral Nerve in Python

**DOI:** 10.1007/s12021-018-9383-z

**Published:** 2018-06-15

**Authors:** Carl H. Lubba, Yann Le Guen, Sarah Jarvis, Nick S. Jones, Simon C. Cork, Amir Eftekhar, Simon R. Schultz

**Affiliations:** 1 Department of Bioengineering, Imperial College London, South Kensington, London, SW7 2AZ UK; 2 Department of Mathematics, Imperial College London, South Kensington, London, SW7 2AZ UK; 3 Department of Medicine, Imperial College London, South Kensington, London, SW7 2AZ UK; 4 Department of Electrical and Electronic Engineering, Imperial College London, South Kensington, London, SW7 2AZ UK

**Keywords:** Simulation, Peripheral nerve, Finite element model, Biophysics, Bioelectronic medicines

## Abstract

Bioelectronic Medicines that modulate the activity patterns on peripheral nerves have promise as a new way of treating diverse medical conditions from epilepsy to rheumatism. Progress in the field builds upon time consuming and expensive experiments in living organisms. To reduce experimentation load and allow for a faster, more detailed analysis of peripheral nerve stimulation and recording, computational models incorporating experimental insights will be of great help. We present a peripheral nerve simulator that combines biophysical axon models and numerically solved and idealised extracellular space models in one environment. We modelled the extracellular space as a three-dimensional resistive continuum governed by the electro-quasistatic approximation of the Maxwell equations. Potential distributions were precomputed in finite element models for different media (homogeneous, nerve in saline, nerve in cuff) and imported into our simulator. Axons, on the other hand, were modelled more abstractly as one-dimensional chains of compartments. Unmyelinated fibres were based on the Hodgkin-Huxley model; for myelinated fibres, we adapted the model proposed by McIntyre et al. in 2002 to smaller diameters. To obtain realistic axon shapes, an iterative algorithm positioned fibres along the nerve with a variable tortuosity fit to imaged trajectories. We validated our model with data from the stimulated rat vagus nerve. Simulation results predicted that tortuosity alters recorded signal shapes and increases stimulation thresholds. The model we developed can easily be adapted to different nerves, and may be of use for Bioelectronic Medicine research in the future.

## Introduction

Manipulations of the peripheral nervous system (PNS) by implanted devices might soon serve as a treatment for various medical conditions. Such Bioelectronic Medicines (Birmingham et al. 2014) can be seen as a permanent, highly localised alternative to molecular medicines with less side effects. Already today, vagus nerve stimulation is being applied in patients suffering from refractory epilepsy (Milby et al. 2010), Alzheimer’s disease (Sjogren et al. 1997), anxiety (George et al. 2008), obesity (Krzysztof et al. 2011), chronic heart failure (Rousselet et al. 2014), and to evoke anti-inflammatory effects (Meregnani et al. 2011; Borovikova et al. 2000). The more localised targeting of organs e.g. of the heart (Pohl et al. 2015) has also shown promising results in animal experiments.

Current devices operate in open-loop mode and stimulation selectivity is low. Future Bioelectronic Medicines will need more precise stimulation interfaces and the capability to analyse (or ‘decode’) nerve activity to stimulate in an adaptive manner. First advances towards a decoding of information from peripheral nerves have been successfully undertaken (Citi et al. 2008; Lubba et al. 2017). To both accelerate the design of interfaces and to further develop decoding algorithms, computational peripheral nerve models that integrate physiological insights acquired in experiments at different levels (i.e. properties of axons, extracellular media, spontaneous activity patterns, organ responses) will be of great merit to predict stimulation efficiency and recording selectivity and as a source of surrogate data.

Previous efforts to simulate peripheral nerves date back approximately twenty years. In 1997, Struijk (1997) developed a 2D model of recordings from myelinated peripheral axons. Models for stimulation were also proposed at the time (Veltink et al. 1989; Goodall et al. 1995). One main difficulty in peripheral nerve simulations, already appreciated at that time, is the calculation of extracellular potentials from membrane currents in the inhomogeneous medium surrounding the axons. As a major difference from recordings in the central nervous system (CNS), the recording method (e.g. cuff electrode, oil bath) often shapes nature of the extracellular space in the PNS. Early simulations therefore often concentrated on modelling the extracellular medium whilst approximating axons with simplified models such as the Fitz-Hugh-Nagumo equations (Plachta et al. 2012, e.g.) or the McNeal model (McNeal 1976; Veltink et al. 1989). Only recently precise biophysical axon models and detailed, numerically solved models of the surrounding medium have been combined (Grinberg et al. 2008; Raspopovic et al. 2011), thanks to increasing availability of computational resources.

For the general task of modelling extracellular potentials caused by cells (axons), many choices at different levels of detail and computational cost exist. The most simple approach is based on volume conductor theory: the extracellular space is modelled as being resistive, homogeneous, and isotropic (Holt and Koch 1999; Lindén et al. 2014) so that the extracellular potential becomes an analytic function of source (membrane) currents. The latter are obtained from compartmental simulations of the cell membrane in which usually the extracellular potentials are assumed to be constant. However, in peripheral nerves, the surrounding medium is not homogeneous or isotropic, requiring a more complex approach. To accommodate conductivity inhomogeneities, precomputed membrane currents from compartmental cell simulators can be imported into a finite element model (FEM) solver (as a point source or boundary condition) where the potential over the entire space and time span is computed based on the quasistatic Maxwell equations (cf. McIntyre and Grill 2001; Lempka and McIntyre 2013; Ness et al. 2015). This costlier method was employed in the recent aforementioned works on peripheral nerves (Grinberg et al. 2008; Raspopovic et al. 2011). It has disadvantages, however. The model needs to be defined in two different environments, the compartmental axon simulator and the FEM solver. Both environments need to be coordinated in terms of geometry, coordinate systems, units, and so on and setting up a model this way is a time consuming and error prone process. There exist commercial simulation solutions that combine a compartmental simulator and a FEM solver in a single framework, e.g. Sim4Life (Zurich MedTech AG) but no openly available simulators. As a further limitation of current hybrid solutions, the FEM simulation is run for each point in time even though the quasistatic approximation of the Maxwell equations allows for a separation of time and space. Static FEM simulations for each source position are sufficient (see Methods).

More detailed models that go beyond the simplifying assumptions of a constant extracellular potential during axon simulation and a quasistatic extracellular space were proposed as well. In particular, electrical feedback of neuronal activity on membrane processes, ephaptic coupling, is neglected by the methods mentioned so far. However, it can be significant (Tveito et al. 2017; Bokil et al. 2001). To incorporate such feedback, the entire model (including the intracellular space and membranes) can be simulated in a FEM solver as in Agudelo-Toro and Neef (2013) and Tveito et al. (2017). Even if elegantly capturing intra-, extracellular, and membrane effects in a combined, self-contained model, the calculation becomes more expensive and is only suitable for simple geometries (Tveito et al. 2017). An even higher degree of accuracy can be attained by going beyond the quasistatic Maxwell equations and incorporating electrodiffusive effects (diffusion of charge ions) in Poisson-Nernst-Planck solvers, see Pods et al. (2013) and Halnes et al. (2016) or the simplified version (electroneutral model) (Pods 2017). Whilst offering great degree of detail, the computation becomes very expensive in those formulations.

In face of those choices, our open-source toolbox PyPNS^1^ aims at realising an compromise between usability, computational efficiency, and accuracy for a bundle of many axons. Our approach is in principle very comparable to a model that precomputes membrane currents in a compartmental simulator and imports them into an FEM simulation. We use the NEURON simulator (Hines and Carnevale 1997) to model axon membrane processes at the scale of ion channels. Standard models for both myelinated and unmyelinated axons in the diameter range found in the periphery were implemented. The extracellular space was governed by the electro-quasistatic approximation of the Maxwell equations. Ephaptic coupling and electrodiffusion were neglected for the sake of computational efficiency. Importantly however, PyPNS improves the efficiency and usability of previous approaches by avoiding to run FEM simulations repeatedly for every simulation. Instead, we took advantage of the quasistatic approximation of the Maxwell equations to separate time and space and simplified the nerve geometry to create symmetries. Potential distributions could thereby be precomputed in a reusable way for arbitrary axon shapes and were imported into PyPNS. In this way, the accuracy of hybrid FEM-based solutions was reached at the computational cost of the simple volume conductor method. In addition, PyPNS adds detail compared to previous simulations by letting the user choose the degree of axon tortuosity. Tortuosity is expected to be particularly relevant for peripheral nerves. An increased axon length can act as a buffer against mechanical influences only present in the periphery; this buffer allows curvier axons. Lastly, our simulator is embedded into the Python ecosystem. For the entire simulation, the user can stay in Python to stimulate nerves and record from them in silico.

## Methodology

### Nerve Stimulation Experiments

**Fig. 1:**
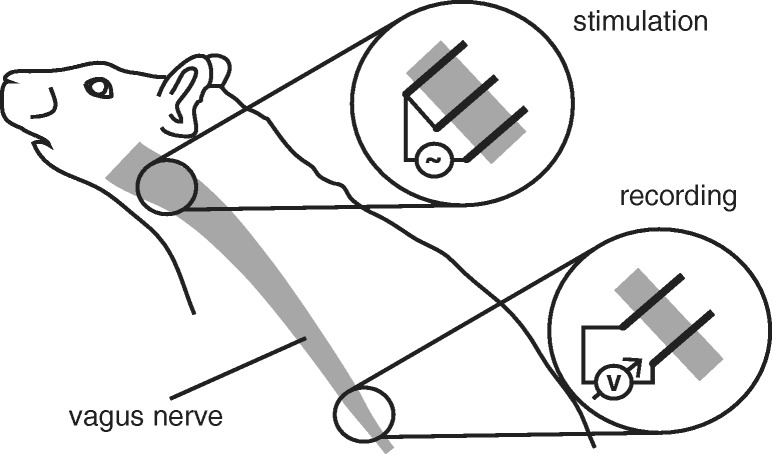
The validation data were obtained through stimulation of a rat vagus nerve. A pseudotripolar electrode excited axons at the cervical vagus nerve, signals were picked up at the subdiaphragmatic vagus nerve with a bipolar electrode

Experiments were carried out in accordance with the Animals (Scientific Procedures) Act 1986 (United Kingdom) and Home Office (United Kingdom) approved project and personal licences, and experiments were approved by the Imperial College Animal Welfare Ethical Review Board under project licence PPL 70/7365. A male Wistar rat (body weight 350–400 g) was initially anaesthetised with isoflurane. Urethane was then slowly administered through a tail vein (20 mg kg^− 1^). The left cervical vagus nerve was exposed and contacted with a stainless steel pseudo-tripolar hook electrode of pole distance 1–2 mm for stimulation. To record from the nerve, a bipolar platinum hook electrode (pole distance 2–3 mm) was then wrapped around the anterior branch of the subdiaphragmatic vagus nerve with an Ag/AgCl ground electrode placed in the abdominal cavity. Distance between recording and stimulating electrodes was 8–10 cm. See Fig. 1. Mineral oil was applied to each site to insulate the electrodes from environmental and proximal noise sources. Stimulation of the cervical vagus nerve was performed using a Keithley 6221 current source, controlled by Standard Commands for Programmable Instruments (SCPI) via a custom built Matlab interface. Bipolar cuff recordings were achieved with an Intan Technology RHD2000 system, using a 16-channel bipolar ended amplifier (RHD221). The obtained recordings were averaged over 10 repeated stimulations in the same animal.

### Imaging of Peripheral Nerve Tortuosity

All procedures were carried out in accordance with the Animals (Scientific Procedures) Act 1986 (United Kingdom) and Home Office (United Kingdom) approved project and personal licences, and experiments were approved by the Imperial College Animal Welfare Ethical Review Board under project licence PPL 70/7355. To reproduce the morphology of axons, we imaged the vagus and sciatic nerves in mice using two photon fluorescence imaging. In the experiment, ChAT-Cre FLEX-VSFP 2.3 mice were euthanised by intraperitoneal overdose of pentobarbital (150 mg kg^− 1^). The pre-thoracic left and right vagus nerves were surgically exposed and 0.5 cm sections were removed and placed in phosphate buffered saline (155.1 mmol NaCl, 2.96 mmol Na2HPO4, 1.05 mmol KH2PO4) adjusted to 8.0 pH with 1 mol NaOH. Sections of the left and right sciatic nerves of between 1 and 2 cm from above the knee were also removed. To prepare for microscopy, the nerves were placed on microscope slides, stretched until straight, and the nerve ends were fixed with super glue. The preparation was covered with PBS. Distortions potentially caused by the stretching of the nerves were assumed to lie within the physiological range of movement-induced deformations the nerve undergoes in the living organism. A commercial 2P microscope was used for imaging (Scientifca, emission blue channel: 475/50 nm, yellow channel 545/55 nm, 511 nm dichroic, Semrock) whilst exciting at 950 nm using a Ti-Sapphire laser (Mai Tai HP, Spectra-Physics).

### PyPNS Overview

Every PyPNS simulation describes one peripheral nerve consisting of an arbitrary number of unmyelinated and myelinated axons, each with a certain diameter and trajectory. Axons can be activated by synaptic input, intra- and/ or extracellular stimulation. For extracellular recordings, electrodes are positioned along the nerve.

**Fig. 2:**
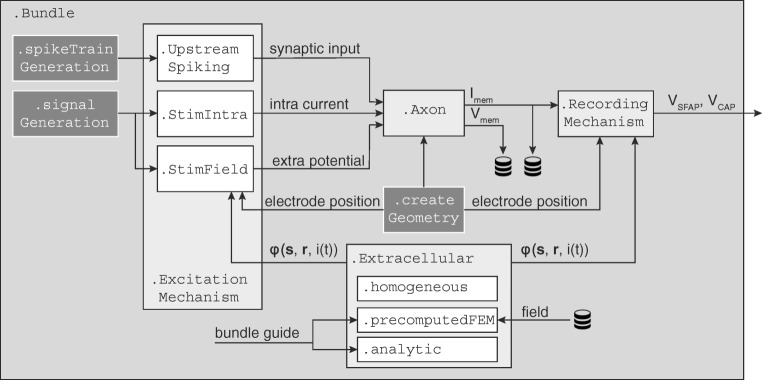
The Axon-class is the central object of PyPNS’s internal information flow. Together with its associated ExcitationMechanism s it defines the NEURON simulation. Extracellular-objects allow the calculation of extracellular potentials given current *i*(*t*), source position **s** and receiver position **r**. They are used by both StimField for extracellular stimulation and by RecordingMechanism for recording. All classes are managed in the Bundle-class and supported by helper modules spikeTrainGeneration, signalGeneration and createGeometry.

The module is organised as several core classes mapped to the physiological entities found in a peripheral nerve (shown in Fig. 2 along with the data flow). All objects are managed by the main class Bundle. This is the central object in the Python domain and represents the whole nerve. It contains instances of the Axon-class that define properties needed by the NEURON simulations. Unmyelinated and Myelinated are derived from the parent Axon-class. Each axon is characterised by its diameter and trajectory. To activate axons, Excitation Mechanism s are added to the Bundle. Those can be either synaptic input (UpstreamSpiking), intracellular stimulation (StimIntra) or extracellular stimulation (StimField). Similarly for recording, electrodes can be added to the whole nerve as a RecordingMechanism. For all interactions with the extracellular space, i.e. extracellular stimulation or recording, a model of the medium defined in a Extracellular-class has to be set. This can be either homogeneous (homogeneous), an FEM result (precomputedFEM) or an analytically defined potential distribution (analytic).

In the simulation step, the definition of each axon in Bundle is sequentially transmitted to NEURON via the Python-NEURON-Interface (Hines et al. 2009) alongside its associated ExcitationMechanism s. After the calculation of single axon membrane processes is finished in NEURON, PyPNS computes the extracellular single fibre action potential (SFAP) for the associated RecordingMechanism s from membrane currents. Once all axons have been processed, their contributions to the overall compound action potential (CAP) are added.

### Assumptions and Simplifications

Several assumptions were required for the computational feasibility and efficiency of our model. Axons were assumed to be independent from each other in their activity (no ephaptic coupling). Properties such as diameter, myelination, and channel densities stayed constant along the axon length. The electro-quasistatic approximation of Maxwell’s equations governed the extracellular space, neglecting magnetic induction:

1 $$ \nabla \times E = -\frac{\partial B}{\partial t} \simeq 0  $$

Further, all media were assumed to be purely resistive, so that all changes in current affected the potentials of the entire space immediately. In Maxwell’s equations this results in neglecting displacement currents:

2 $$ \nabla \times H = J + \frac{\partial D}{\partial t} \simeq J  $$

For the brain and in the considered frequency range, the electro-quasistatic approximation is assumed to be valid (Hämäläinen et al. 1993; Bossetti et al. 2008); previous peripheral nerve simulation studies have built on both quasistatic and purely resistive approximations (Raspopovic et al. 2012; Struijk 1997; Veltink et al. 1989; Goodall et al. 1995). Layers of tissue surrounding the nerve were modelled with a circular symmetry and only one fascicle was considered. Extracellular recordings and stimulation did not take into account the electrode-electrolyte interface (see Cantrell et al. (2008) for its effect on stimulation efficiency).

### Axon Models

**Fig. 3:**
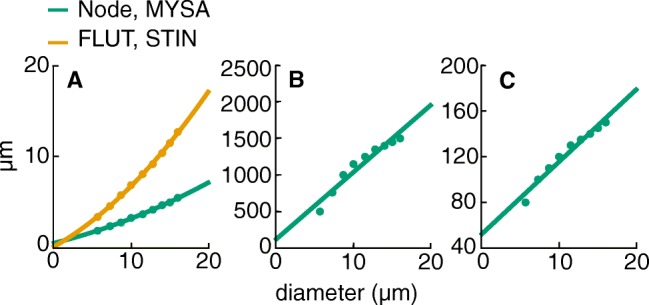
Linear and quadratic fits were used to extrapolate the parameters of myelinated axons to smaller diameters. **a** Diameters of all segments – nodes, MYSA (myelin attachment segments), and paranodal elements FLUT (paranode main segment) and STIN (internode segment, see McIntyre et al. (2002) for more information on the model)–were fit quadratically to prevent negative values. Node distance (**b**) and number of myelin sheaths (**c**) were extrapolated linearly

We used the original Hodgkin-Huxley parameters (Hodgkin and Huxley 1952) for unmyelinated axons. Myelinated ones were based on the model of McIntyre et al. (2002) that has originally been developed for peripheral motor fibres with thicker diameters (5.7–16.0 *μ* m). To match the thinner axons found in the PNS (0.2–3 *μ* m), we extrapolated all diameter dependent parameters to smaller diameters as shown in Fig. 3. Extrapolated parameters were: (1) the diameters of the different segments – nodes, MYSA (myelin attachment segment), FLUT (paranode main segment), STIN (internode segment), (2) node distance and (3) the number of myelin sheaths. Neither model is claimed to exactly match the properties of single neurons found in the PNS. We aimed to implement a generalised framework in which parameters can be fine-tuned to match specific datasets.

### Generation of Axonal Geometry

Axons in peripheral nerves are not perfectly straight, but instead follow the nerve path with a certain degree of tortuosity. To model this in our simulation without defining the geometry for each fibre manually we iteratively placed straight axon segments along a previously defined bundle guide, itself composed of longer straight segments. In each step, the axon segment direction **a**_*i*_ was calculated as

3 $$ \mathbf{a}_{i} = \frac{\mathbf{a}_{i-1} + (1.1-\alpha) \cdot \mathbf{b}_{k} + \alpha \cdot \mathbf{w}_{i}}{||\mathbf{a}_{i-1} + (1.1-\alpha) \cdot \mathbf{b}_{k} + \alpha \cdot \mathbf{w}_{i}||},  $$

based on the corresponding bundle guide segment direction **b**_*k*_ (*k* ≤ *i* as bundle guide segments were longer than axon segments), the previous axon segment direction **a**_*i*− 1_ and a random component perpendicular to the bundle guide segment direction **w**_*i*_. All vectors have unit length. The parameter *α* ∈ [0,1] regulates the tortuosity of the axon and can, together with the distribution of ||**w**||, be fit to geometries measured by microscopy. The factor (1.1 − *α*), rather than (1 − *α*), was chosen to maintain forward axon growth. See Appendix A for the exact implementation of **w**_*i*_ which insures that axons stay within the nerve.

To fit our axon placement method to realistic axon trajectories, fibres in microscopy images were manually traced and segmented into straight sections of length 15 *μ* m. For all traced axons of one nerve, the normalised difference in direction between consecutive segments *c* = ||**a**_*i*_ −**a**_*i*+ 1_|| was calculated. We then compared the *c*-distribution of imaged, traced axons to the ones obtained from artificial fibres placed at different tortuosity coefficients *α* and ||**w**||-distributions to select the best fit. For details see Appendix B.

### Extracellular Potentials

**Fig. 4:**
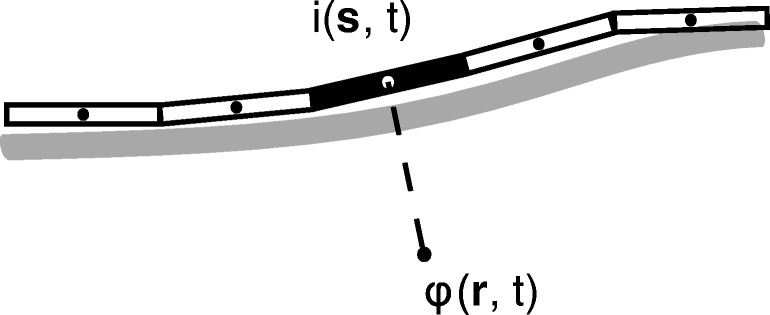
Axon segments can be interpreted as current point sources. The extracellular potential *ϕ*(**r**,*t*) at position **r** caused by a current *i*(**s**,*t*) at position **s** is determined by current time course scaled with a static potential depending on the extracellular space and the spatial relation between source and receiver position

Recordings from peripheral nerves capture changes in the potential of the extracellular medium caused by membrane currents. To calculate those changes in PyPNS, axon segments were interpreted as point current sources, each causing a potential change in the entire medium.^2^ See Fig. 4. Potentials generated by all current sources were superposed. From the electro-quasistatic approximation of the Maxwell equations, combined with pure resistivity, time and space can be separated in the compound action potential (CAP) calculation:

4 $$ \phi_{\text{CAP}}(\mathbf{r}, t) = \sum\limits_{\mathbf{s}_{i}} \,\frac{\phi_{\text{static}}(\mathbf{s}_{i}, \mathbf{r}, I_{\text{ref}})}{I_{\text{ref}}} \cdot i(\mathbf{s}_{i}, t).  $$

The extracellular potential over time at receiver position **r**, *ϕ*_CAP_(**r**,*t*), was calculated as the sum over single axon segment contributions. The contribution of one segment at position **s**_*i*_ to the potential recorded at position **r** was obtained from a known static potential *ϕ*_static_(**s**_*i*_,**r**,*I*_ref_) at reference current *I*_ref_ that was then scaled by the temporally varying membrane current of the segment *i*(**s**_*i*_,*t*).

Extracellular stimulation follows exactly the same principle, with stimulation electrodes modelled as assemblies of point current sources and axon segments as potential receivers.

To further clarify the implications of Eq. 4 on extracellular recordings, consider a single straight axon on the *z*-axis, so that *ϕ*_static_(**s**,**r**,*I*_ref_) becomes *ϕ*_static_(*z*,*I*_ref_) with *z* = (**s** −**r**) ⋅**e**_*z*_. The translation of membrane current to recorded single fibre action potential (SFAP) in the extracellular medium is then solely determined by the profile of the static potential over longitudinal distance:

5 $$ \phi_{\text{SFAP}}(t) = \sum\limits_{z_{i}} \frac{\phi_{\text{static}}(z_{i}, I_{\text{ref}})}{I_{\text{ref}}} \cdot i\left( z_{i}, t\right).  $$

**Fig. 5:**
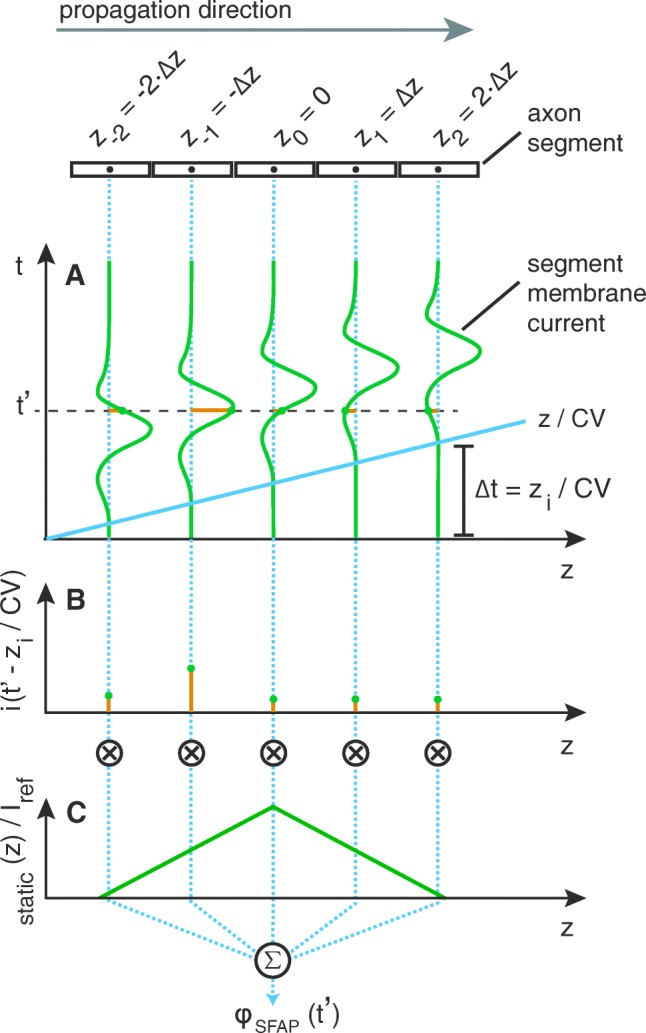
The impact of the longitudinal profile *ϕ*_SFAP_(*z*) on SFAPs can be understood by studying the potential caused by a perfectly straight axon recorded at *z*_0_ = 0 for $t = t^{\prime }$. Axon segments of length Δ*z* exhibit the exact same current time course except for a delay Δ*t* = *z*_*i*_/*CV* (**a**). The potential *ϕ*_SFAP_ at $t = t^{\prime }$ is then obtained as the sum over membrane currents $i(z_{i}\,| \, t = t^{\prime })$ shown in (**b**), multiplied by the static potential *ϕ*_static_(*z*_*i*_,*I*_ref_)/*I*_ref_ (**c**)

As Fig. 5 demonstrates, the membrane current of each axon segment is temporally displaced according to its distance *z*_*i*_ and the conduction velocity *CV* (Fig. 5a):

6 $$ i(z_{i}, t) = i\left( t - \frac{z_{i}}{CV}\,| \, z = 0\right) := i_{0}\left( t - \frac{z_{i}}{CV}\right).  $$

For one *t* = *t*^′^, the instantaneous currents *i*(*z*_*i*_ | *t* = *t*^′^) = *i*_0_(*t*^′^− *z*_*i*_/*CV* ) of all segments shown in Fig. 5b are multiplied by the static potential corresponding to their spatial displacement (Fig. 5c) and added up.

If one assumes, as an extreme example, the Kronecker delta as a profile (*ϕ*(*z*) = *δ*(*z*)), the SFAP would have exactly the same time course as the membrane current. On the other hand a constant profile *ϕ*(*z*) = *c* will make the resulting SFAP vanish because of charge conservation ($\int i(t) dt = 0 \Rightarrow \int i(z/CV) dz/CV = 0$). The recorded action potential is maximal if positive and negative peaks of membrane current add up constructively. To quantify when this happens, an active length *l*_a_ of an axon can be defined as

7 $$ l_{\text{a}} = t_{\text{a}} \cdot CV,  $$

with *t*_a_ denoting the time during which an axon segment emits current of constant sign and *CV* the conduction velocity. Membrane current is of the same sign over length *l*_a_. The match between this length and the range of the profile (Δ*z* = *z*_2_ − *z*_1_ with *ϕ*(*z*) > 0 for *z* in [*z*_1_,*z*_2_]) will determine the amplitude of the SFAP – in addition to a scaling factor depending on the absolute values of *ϕ*_static_(*z*) in Eq. 4.

### Homogeneous Media

If the medium is assumed to be homogeneous with a constant conductivity *σ*, the potential *ϕ*(**r**,*t*) at **r** caused by a point source of current *i*(**s**,*t*) at **s** can be analytically written (see Malmivuo and Plonsey 1995, Chapter 8 or Lindén et al. 2014 for reference) as

8 $$ \phi(\mathbf{r}, t) = \frac{1}{4 \pi \sigma}\frac{i(\mathbf{s},t)}{|\mathbf{s} - \mathbf{r}|} .  $$

Compared to the formulation in Eq. 4, the static potential term that translates current to voltage here became

9 $$ \frac{\phi_{\text{static}}(\mathbf{s}, \mathbf{r}, I_{\text{ref}})}{I_{\text{ref}}} = \frac{1}{4 \pi \sigma |\mathbf{s} - \mathbf{r}|}.  $$

PyPNS implements the homogeneous case as PyPNS.Extracellular.homogeneous.

### Radially Inhomogeneous Media

**Fig. 6:**
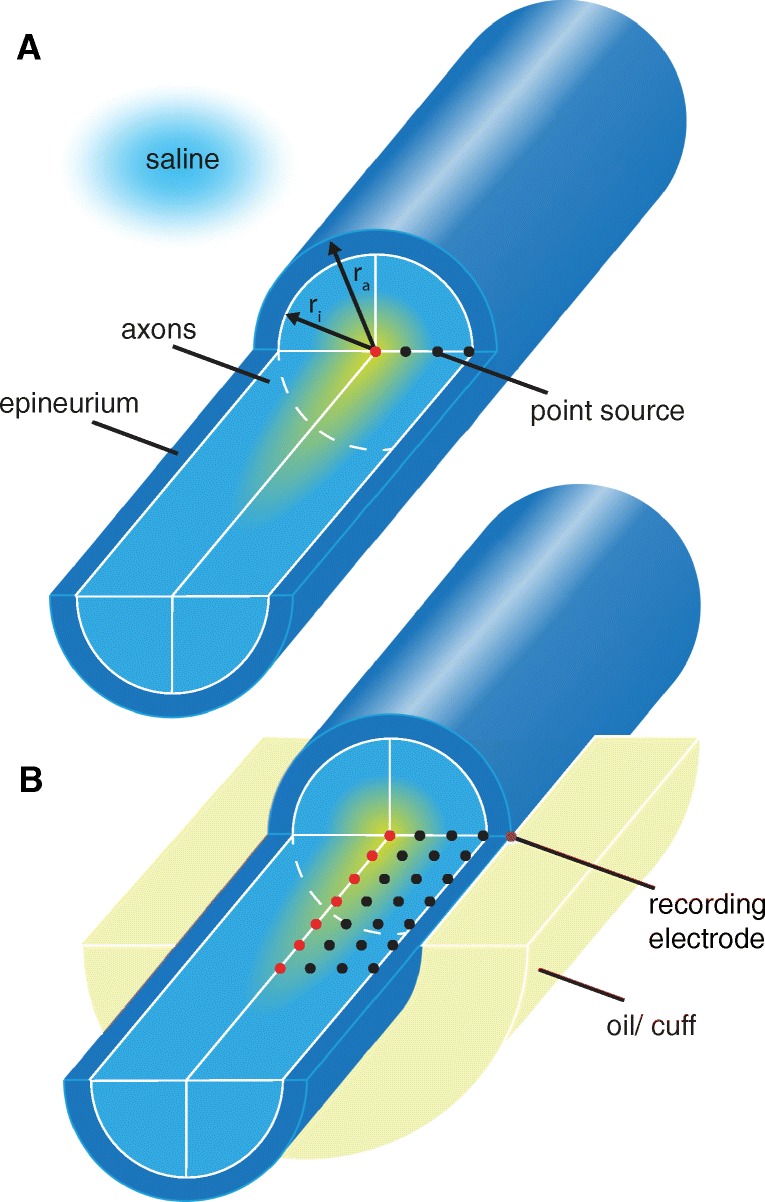
A circularly symmetric geometry makes it possible to import precomputed potential fields. The nerve is modelled as axons (white matter) surrounded by the epineurium. The positions of exemplary current point sources, each generating one potential field, are shown. For radially inhomogeneous media, a line of sources does characterise all unique fields. For longitudinal inhomogeneities (**a**), potential fields for a two-dimensional array of point current sources need to be precomputed (**b**)

As the medium surrounding the axons in peripheral nerves is anisotropic and inhomogeneous, the homogeneous assumption is not appropriate. Consequently, no exact analytical solution for the potential caused by a point current source exists and numerical methods become necessary.^3^ In order to reduce computational load, we precomputed potential fields once in a finite element model (FEM) and then imported and reused them in PyPNS. This means that the computationally expensive field calculation only had to be carried out once per extracellular medium geometry. To insure the feasibility of this approach, the extracellular space was modelled using the simplified geometry shown in Fig. 6a, with conductivities set to the values given in Table 1. By making the conductivity a function of radius only (i.e. conductivity boundaries were circularly symmetric), a very limited number of unique point source positions exists, each for a different radius (dots in Fig. 6a). We refer to this setup as a radially inhomogeneous medium.

In the FEM solver COMSOL 4.3, the nerve had a length of 10 cm and was placed in a cubic volume of equal edge length. The inner nerve radius was set to 190 *μ* m, the endoneurium thickness to 50 *μ* m. All inner boundaries had von Neumann boundary conditions, the potential of the outer border of the cubic volume was set to zero (Dirichlet boundary condition). The current entered the mesh at a single point.

Voltage fields *ϕ*(*x*,*y*,*z*,*r*) for different radial point source displacements *r* were computed. Due to our assumptions concerning the medium, steady state simulations were sufficient (separation of time and space). The static voltage fields were exported on a grid of *x* ∈−[1.5,1.5] mm with a step of 0.015 mm, *y* ∈ [0, 1.5 mm] with a step of 0.015 mm, *z* ∈ [0,30] mm with a step size of 0.03 mm where *z* is the longitudinal nerve axis and source positions are displaced along *x*. The fields were imported in PyPNS as a linear 4D spline interpolator. PyPNS afterwards scales the static potentials with current time courses as given in Eq. 4 with *I*_ref_ set to 1 nA in COMSOL. The corresponding mechanism in PyPNS is PyPNS.Extracellular.precomputedFEM. When using an imported potential field, attention has to be paid to the source radii used in the FEM precomputation step. The radius selected in PyPNS needs to lie within the precomputed range. E.g. for stimulation, radii might be larger than the nerve radius whereas for recording the precomputed source radii have to lie within the nerve. Of course, different precomputed fields can be used for recording and stimulation respectively.

**Table 1:** Conductivity of different tissues contained in the simulated peripheral nerve; colours correspond to Fig. 6 (Capogrosso et al. 2013; Struijk 1997)

Tissue	Conductivity S m^− 1^
Axons (light blue)	0.5 longitudinal, 0.8 transversal
Epineurium (darker blue)	0.1 isotropic
Saline (white)	2.0 isotropic

### Longitudinally Inhomogeneous Media

In electrophysiological experiments, the nerve does not usually lie within its natural surrounding tissue. Instead, to improve stimulation and recording performance, a cuff or a mineral oil bath increases the extracellular resistivity. The medium is in this case no longer longitudinally homogeneous, and any longitudinal shift in current source position will result in a different potential field. For stimulation, the current source (stimulation electrode) position can be fixed and the precomputation of very few potential fields, each for one electrode radius, characterises the effect of the electrode completely. For recordings, however, the longitudinal source position necessarily varies, as the axon segments extend through the nerve. Therefore, to cover all unique axon segment potential fields, a 2D-array of source positions distributed along both radial and longitudinal direction must be precomputed, as shown in Fig. 6b.

Note that without circular symmetry, a volume of source positions would need to be simulated, making the precomputation infeasible.^4^ In this case, the most efficient approach would be to fix the axon geometries for one particular case, perform an FEM simulation for each axon segment position and either export the potential fields for the whole space or also fix the electrode positions and export the potentials only at the electrodes. This method, however, is less universal, much more computationally expensive, and involves a lot more coordination between FEM simulation and compartmental axon model.

We found that for recording, a reasonable number of current source positions (∼ 20, each using about 40MB of memory) could not abolish interpolation errors between fields from longitudinally adjacent source positions, causing artefacts in the extracellular action potentials. To generate recordings without artefacts, a smoothed transfer function between point current source position and potential in the cuff was fit to FEM model results. Details are given in Appendix C. This transfer function served in PyPNS as a variant of PyPNS.Extracellular.analytic.

## Results

### Axon Models

For thin (< 1 *μ* m) myelinated axons, extrapolated parameters from the McIntyre model (McIntyre et al. 2002) yielded bursting behaviour as soon as the fibres were activated through either synaptic input or stimulation. To prevent this, the potassium channel density at the nodes was increased by a factor of 1.5. Node size reduction with diameter achieved the same effect but is not observed (Tuisku and Hildebrand 1992; Berthold and Rydmark 1983). Potassium channels in the paranodal regions (not included in the original model) have been observed physiologically (Poliak and Peles 2003; Röper and Schwarz 1989) but their integration in the model could not abolish bursting. Myelinated conduction velocity (CV) fit experimental data well (CV [ms^− 1^] ∼ 5 ⋅ *d* with diameter *d* in *μ* m). Unmyelinated axons based on Hodgkin-Huxley channels had very low conduction velocities, CV $\sim 0.4 \cdot \sqrt {d}$, in comparison with expected values of around $2 \cdot \sqrt {d}$ (Waxman 1980). This is an inherent property of the Hodgkin-Huxley axon model.

**Fig. 7:**
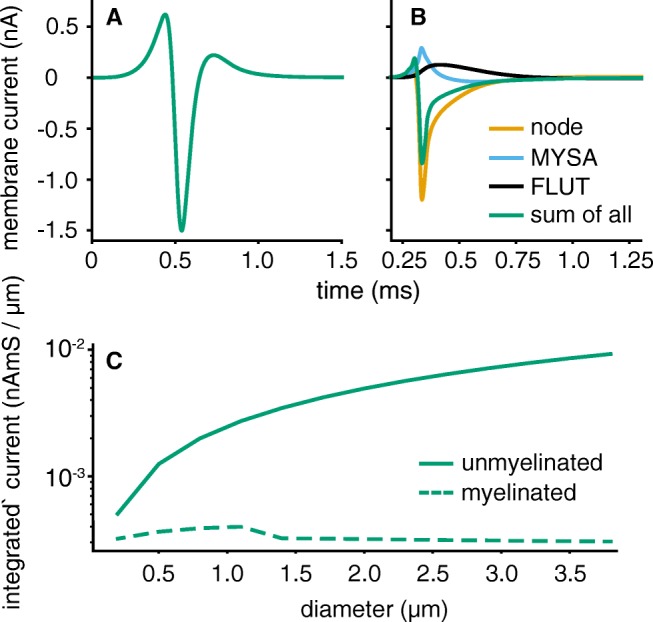
Unmyelinated axons (**a**) produce a smoother membrane time course than myelinated (**b**) ones. Both axons had a diameter of 3 *μ* m. **c** Unmyelinated axons produce a higher current output per distance. The integrated absolute current during a single action potential over axon length is shown

As membrane current directly shapes extracellular potential recordings, Fig. 7 compares the membrane current in time for one unmyelinated axon segment (Fig. 7a) and one node of Ranvier (Fig. 7b). In Fig. 7c, the integrated current output is plotted over diameters. Importantly, unmyelinated axons emitted more current per distance and the signal shapes differed considerably. The unmyelinated current time course was smooth, whereas the myelinated one was more complex with a sharp peak and a long lasting recovery. The latter axons contain different segments (node, myelin attachment segment (MYSA), paranodal main segments (FLUT)) which all contribute to the overall current output and thereby caused the more complex shape. See the model of McIntyre et al. (2002) for more details on section types.

### Profiles of Extracellular Media

**Fig. 8:**
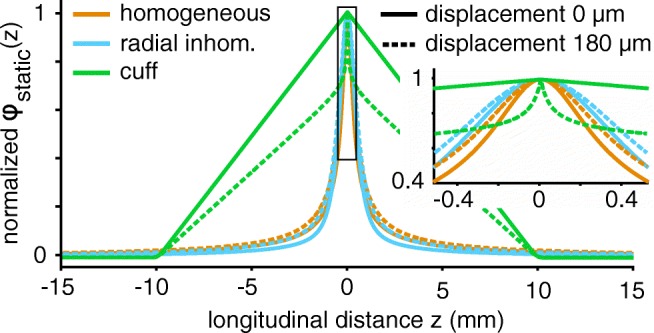
Compared to the homogeneous and radially inhomogeneous extracellular media the cuff insulation caused a much softer and strikingly linear characteristic. Electrode radius was 235 *μ* m. The profiles are shown for two radial axon displacements in solid and dashed lines respectively

In “Extracellular Potentials” we described the impact of the longitudinal profile *ϕ*_static_(*z*) on the single fibre action potentials (SFAPs). Building on these considerations, the normalised *ϕ*_static_(*z*)-profiles of our media can be compared. Figure 8 shows the normalised static potentials over distance for all three media and makes the strong impact of the cuff insulation obvious. The potential profile became smooth, stretched out in space. The thin nerve surrounded by an insulation acted as two parallel resistors, causing a linear characteristic. For radial displacements of the current source towards the electrode, a sharp peak emerged (see also Fig. 18). We expect fast conducting axons with long active length *l*_a_ to best match this large range profile. The other two media had a different, much narrower characteristic. Radial inhomogeneities produced a slightly smoother potential profile compared to the homogeneous medium but differences remained small. Both profiles decayed a lot steeper with longitudinal distance than in the cuff and were therefore expected to better suit slower conducting axons with a shorter *l*_a_.

### Extracellular Single Fibre Action Potentials

**Fig. 9:**
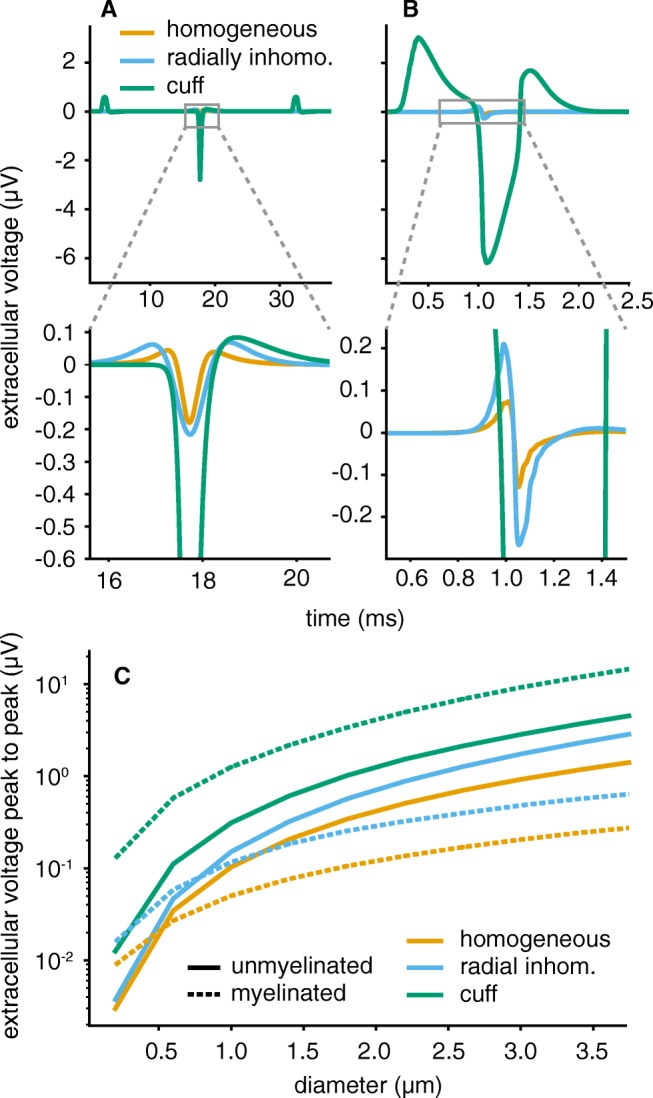
Unmyelinated and myelinated SFAPs showed different sensitivities towards the extracellular space. In the upper plots (**a**, **b**), diameters were set to 3 *μ* m. **a** The main peak of unmyelinated fibres mostly varied in amplitude over media, not in shape. In cuff insulated nerves, additional side peaks emerged. **b** Myelinated fibres produced much higher and longer lasting SFAPs in the cuff insulated medium. Both axons had diameter 3 *μ* m, were placed centrally within the nerve and recorded by a circular monopolar electrode with radius 235 *μ* m. Conductivity of the homogeneous medium was set to 1 S m^− 1^. Lower row shows zoomed-in plots. **c** The amplitude boost achieved by cuff insulation was stronger for myelinated than for unmyelinated axons over the whole diameter range. For the other two media, unmyelinated SFAPs produced stronger SFAP amplitudes at diameters above 0.5 and 1 *μ* m respectively

In Fig. 9, the effect of different extracellular media on the resulting SFAPs can be compared. The axons were activated by intracellular stimulation and recorded with a monopolar circular electrode at radius 235 *μ* m.^5^ In the cuff medium, the electrode was placed centrally as shown for one point electrode in Fig. 6b. Figure 9a shows extracellular potentials from a single unmyelinated fibre. Between the three different media, mostly amplitude varied with only slight differences in shape. Insulating the nerve with a cuff increased the potential by a factor of about ten and caused a narrower signal shape. In addition, an entrance and an exit peak at the sides of the cuff arose that were not present in the two longitudinally homogeneous media. The radially inhomogeneous medium slightly stretched the action potential in time which can be explained by the preference of current to flow along the nerve rather than transversally (compare to profile in Fig. 8).

The SFAP of myelinated axons in Fig. 9b was much more strongly affected than the unmyelinated fibres when insulating the nerve. Whilst the difference between homogeneous and radially inhomogeneous medium remained small, myelinated SFAP amplitude increased by a factor of about 20 in the cuff and shape was changed radically. The recorded signal lasted longer and had (as for unmyelinated axons) a negative main and two positive entrance and exit peaks.

Figure 9c compares the SFAP amplitude for unmyelinated and myelinated axons over diameters and media. Whilst the SFAP amplitude of unmyelinated axons was similar and even higher than myelinated SFAPs in homogeneous and radially inhomogeneous media, myelinated fibres achieved much stronger amplitudes following cuff insulation – even though their membrane current output is substantially lower compared to unmyelinated axons (see Fig. 7). This difference in reaction to the cuff medium between fibre types can be explained by the two different mechanisms through which cuff insulation changed SFAP amplitude. The first one is the increased extracellular resistance. Current cannot freely dissipate into the surrounding tissue but needs to flow along the thin nerve. As membrane current was modelled to be independent of the medium, an increase in extracellular resistance equaled an increase in extracellular potential. This effect increases SFAP amplitude equally for both fibre types. The second one – that can explain the difference in amplitude gain between fibre types – is the match of active length (as defined in Eq. 7) and cuff dimension (equal to range of the profile; 20 mm in this case) as detailed in “Extracellular Potentials”. For a myelinated axon of diameter 3 *μ* m the active length evaluated to approximately 0.5 ms ⋅ 15 ms^− 1^ = 7.5 mm, an unmyelinated axon of this diameter only had an active length of about 0.5 ms ⋅ 1 ms^− 1^ = 0.5 mm. Figure 9c demonstrates the matching effect between myelinated axons and the cuff over all diameters.

### Effects of Varying the Cuff Length

**Fig. 10:**
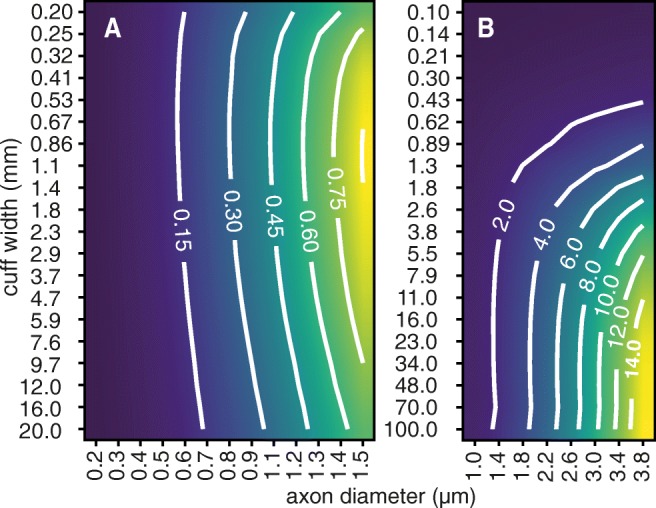
Unmyelinated and myelinated axons have different ideal cuff lengths. For unmyelinated fibres (**a**), very short ranges around 1 mm produce the maximal amplitude. For myelinated ones (**b**), the amplitude only rises with length. Contour lines show the peak-to-peak amplitude in *μ* V

As a tool for Bioelectronic Medicines, PyPNS should help the design of peripheral nerve interfaces. Here we take a look at the impact of cuff electrode length on the recorded signal amplitude. Figure 10 demonstrates how unmyelinated and myelinated fibres require different cuff lengths for a maximal SFAP amplitude. Unmyelinated fibres with their lower conduction velocity and therefore shorter active length produce the strongest signals for (theoretic) cuff lengths of about 1 mm. Whilst those are most likely not achievable, medium lengths of about 1 cm seem reasonable according to our simulation. The amplitude of myelinated axons keeps rising until the investigated maximum cuff length of 10 cm but starts saturating at about 1 cm. PyPNS therefore predicts an ideal cuff length in this order. Results will vary for a more accurate unmyelinated axon model, where higher conduction velocities would be expected to increase the ideal cuff length.

### Compound Action Potentials

For validation, we aimed at reproducing experimental recordings from the stimulated rat vagus nerve in PyPNS. To this end we obtained diameter distributions and fibre counts from microscopy images (Prechtl and Powley 1990) as summarised in Table 2 and set the geometry of the nerve and the recording electrodes so as to match the experimental set-up. Outer and inner radius were set to 240 *μ* m and 190 *μ* m respectively; a circular bipolar electrode of radius 235 *μ* m and pole distance 3 mm (20 recording positions per pole) surrounded the nerve. Axons were placed centrally and were activated intracellularly; due to the difference in stimulation threshold between fibres types, the entire population of myelinated and only a small fraction of unmyelinated axons (∼ 20% of 10,000) was triggered. As unmyelinated fibres based on Hodgkin-Huxley channels had very low conduction velocities, we corrected their SFAP timings. The nerve was insulated with mineral oil in the experimental recording. Therefore only the cuff medium should produce similar extracellular signals in the simulation. The results from homogeneous and radially inhomogeneous media are presented as well in the following for comparison.

**Table 2:** Axon number and properties set in the simulation for comparing model results with experimental recordings

Type	#	diameter (*μ* m)
Unmyelinated	2000	∈ (0.2, 1.52) distribution from Prechtl and Powley (1990)
Myelinated	200	$\sim \mathcal {N}(1.7, 0.4)$ (Prechtl and Powley 1990)

**Fig. 11:**
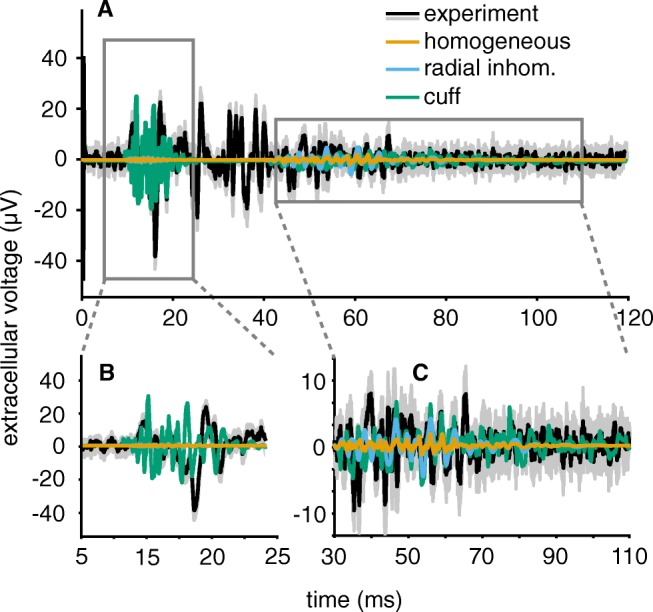
**a** The simulated compound action potential in the cuff medium approaches the experimental recording well in the relevant signal segments. As expected, homogeneous and radially inhomogeneous media lead to much weaker signal amplitudes. For the experimental recording, the grey underlying area indicates the standard deviation over the 10 stimulation repetitions. See Table 2 for axon properties. Distance between stimulation site and bipolar electrode (3 mm pole distance, 235 *μ* m radius) was 8 cm. All axons were activated by intracellular stimulation. The timing of unmyelinated SFAPs was adapted to regular conduction velocity values assumed in mammalian peripheral nerves (CV $= 1.4 \cdot \sqrt {d}$, CV in ms^− 1^, d in *μ* m). **b** The signal from myelinated fibres, which arrive first, appears similar to the experiment. **c** The unmyelinated signal segment matches the amplitude and duration of the experimental recording as well. The signal-to-noise ratio of the recordings is much worse for unmyelinated fibres, however, as the amplitude of their SFAPs is low

Figure 11 plots simulation results in all media against the experimental data and demonstrates a reasonable agreement between simulation and experiment in the time domain. This match naturally only held for the cuff insulated medium – homogeneous and radially inhomogeneous media led to very low extracellular potential amplitudes as expected from their lower tissue resistance. The signal segment between A- and C-fibres from 25 to 40 ms can be attributed to B-fibres and was not compared to the simulation as PyPNS only models A- and C-fibres.

**Table 3:** Quantitative comparison between compound action potentials from experiment and simulation (cuff medium)

Feature	Experiment	Simulation
Myelinated axons		
Area (*μ* V ms)	115	83.2
Peak-to-peak voltage (*μ* V)	57.5	50.3
Zero crossings	50	45
Unmyelinated axons		
Area (*μ* V ms)	147	93.9
Peak-to-peak voltage (*μ* V)	25.2	14.0
Zero crossings	271	133

Especially the signal portion caused by myelinated fibres (Fig. 11b) matches the experiment well in peak amplitudes, area, zero crossings and overall duration. See Table 3 for a quantitative comparison. Unmyelinated axons (Fig. 11c) also produced a CAP comparable to the experiment in both amplitude and timing although the comparison is more difficult as the signal to noise ratio in the experimental data is lower for unmyelinated than for myelinated fibres. Table 3 summarises how area and amplitude of the experimental recording are larger than in the simulation and that there occur considerably more zero crossings in the experiment. The noise present in the experiment will be accountable for a share of those crossings. Of course, the Hodgkin-Huxley model of the unmyelinated axons did not to exactly match the properties of the rat vagus nerve C-fibres, therefore differences in the extracellular recordings were expected.

**Fig. 12:**
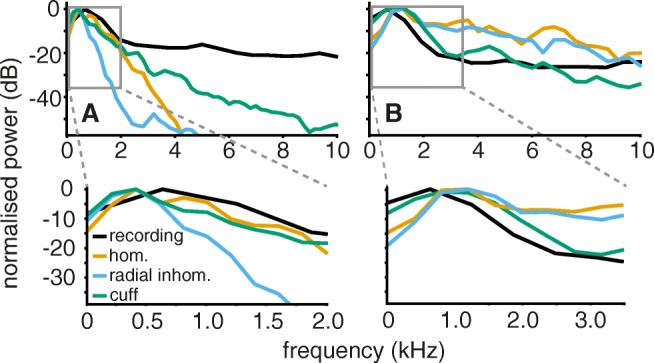
In the frequency domain, simulation and experiment did not match equally well for both fibre types. **a** For unmyelinated axons, the simulation did not perfectly approach the experimental spectrum in any medium with best results for the cuff. **b** The simulated frequency characteristic of myelinated axons in the cuff insulated medium was close to reality

In Fig. 12 simulation and experiment can be compared in the frequency domain for both fibre types. The similarity between simulated and experimental data was comparable to the match in time domain for both myelinated and unmyelinated fibres. The spectrum of the unmyelinated signal proportion in our experimental data (black lines in Fig. 12a) had an overall flat profile with a main peak (lower plot) at around 500 Hz. This characteristic was approached to a certain extent by our model. The spectra in all three media have slighly earlier peaks below 500 Hz but homogeneous and cuff medium result followed the characteristic of the experiment well between 0 and 2 kHz before decaying further below − 20 dB from there. We surmise that the high frequency content of the experimental data may be be caused by high frequency noise from the recording process. Meaningful, spike-event related signal components from experimental recordings usually stay below 2 kHz (Diedrich et al. 2003).

The experimental spectrum of myelinated fibres (Fig. 12b) was dominated by low frequency power below 2 kHz with a peak at about 500 Hz. Our simulation result in the cuff medium matched this characteristic for frequencies over the whole frequency range, although showing a later peak around 1 kHz. The other two media led to a flat characteristic with a larger amount of high frequency power and less low frequency power. This could be predicted from the SFAPs in Fig. 9 where the myelinated SFAP is much wider in the cuff than in the other media.

In conclusion, the experimentally obtained frequency characteristic of both axon types was reasonably matched by our simulation for the cuff medium.

### Fitting Axon Tortuosity to Experimental Data

**Fig. 13:**
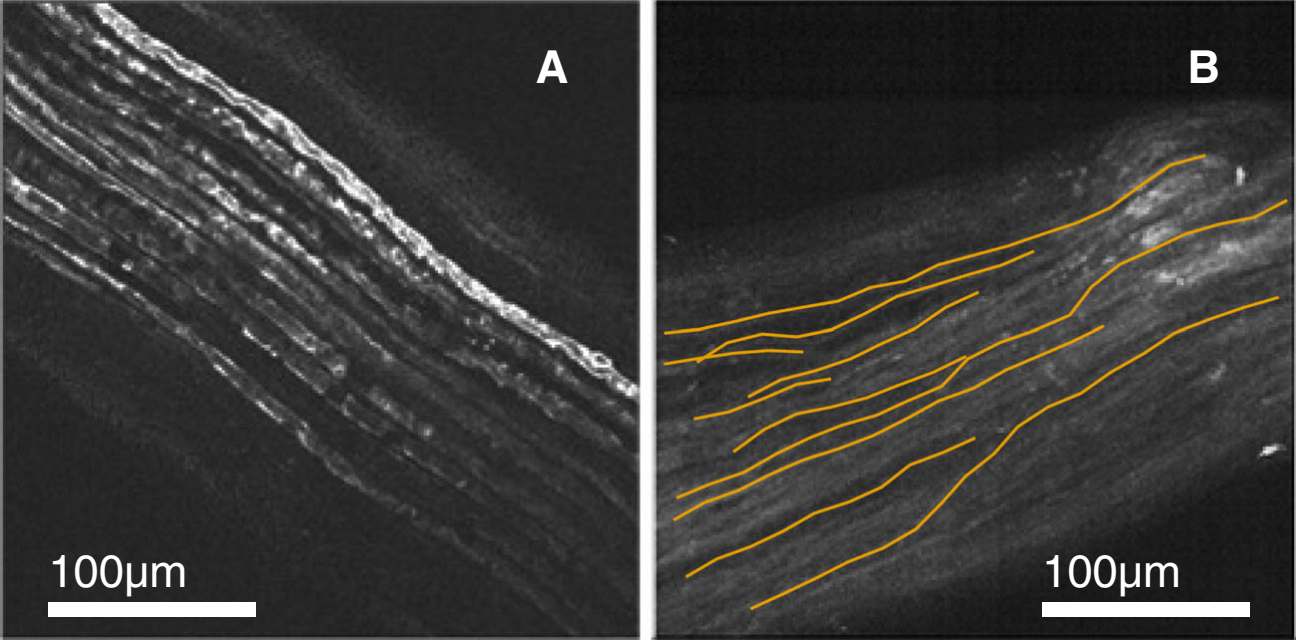
Fluorescence microscopy images of the mouse sciatic and vagus nerve both show slight tortuosity in their axon trajectories. **a** The thick myelinated fibres in the sciatic nerve appear very parallel. **b** The thinner axons in the vagus take a more curvy trajectory. Several manually traced fibres used to fit the model are highlighted in orange

In order to obtain axon shapes close to reality, we compared the distributions of axon segment direction changes *c* as detailed in methods “Imaging of Peripheral Nerve Tortuosity” for imaged mouse sciatic and vagus nerve. See Fig. 13 for fluorescence microscopy images and traced axons.

**Fig. 14:**
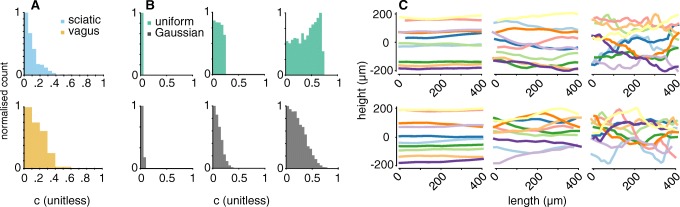
The axon placing algorithm result (**b**, **c**) was fit to tortuosity of microscopy imaged fibres (**a**). **a** Direction change distributions (*c*-distributions) for vagus and sciatic nerve. **b** *c*-distributions in the simulation for both normally and uniformly distributed amplitude of the random component ||**w**|| in Eq. 3 for *α* s of 0.2, 0.6, and 1.0. **c** Example axon trajectories in space for uniform (upper) and Gaussian (lower) ||**w**||-distributions at *α*-values of 0.2, 0.6, and 1.0

In Fig. 14, the obtained direction change distributions from microscopy (Fig. 14a) are compared to the ones of simulated axons (Fig. 14b) alongside a few example axons in space (Fig. 14c). In Fig. 14a, the higher tortuosity observed in the vagus nerve is visible from the wider distribution of segment direction changes (*c*-values) compared to the sciatic nerve. A set of direction change distribution obtained at different parameters (||**w**||-distribution and *α*) in PyPNS is shown in Fig. 14b. When comparing to Fig. 14a, a Gaussian ||**w**||-distribution produced *c*-distributions the most similar to microscopy data. The sciatic nerve then corresponded to an *α*-value of about 0.6, the vagus nerve had a wider *c*-distribution as its axons were curvier, corresponding to a higher *α*. When comparing the trajectories in Fig. 14c from uniform (upper plot) and Gaussian (lower plot) *c*-distributions, it can be seen how the normal distribution of random vector length ||**w**|| leads to both a slightly smoother trajectory and rare strong direction changes, especially for high *α*-values.

### Recording from Tortuous Axons

**Fig. 15:**
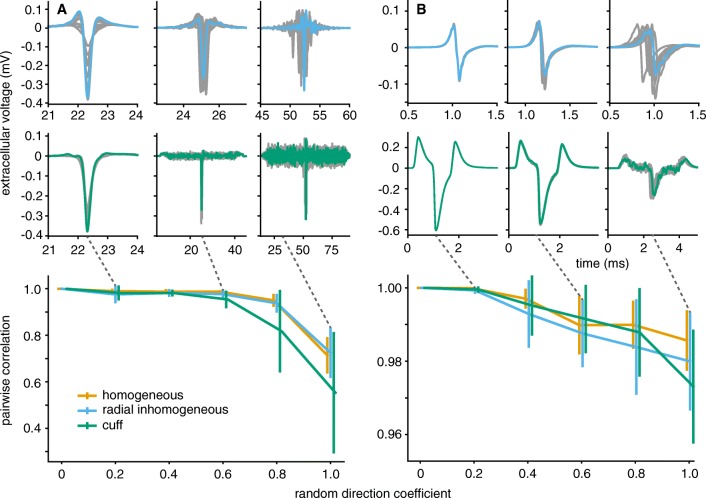
Unmyelinated axons were more sensitive to tortuousity in their SFAP shapes than myelinated ones. Tortuosity parameter *α* was set to 0.2, 0.6, and 1.0 for the signals shown in the first two rows. Grey lines correspond to SFAPs of different trials (axon geometries) at the same parameters. **a** Unmyelinated axons produced SFAPs differing both in timing and shape for the non-insulated nerve (radially inhomogeneous medium), even for small *α*-values of 0.2. In the cuff-insulated nerve (middle row) their signals became noisy at low *α*-values and the main SFAP peak almost disappeared for *α* = 1.0. **b** Myelinated axons mostly differed in timing in the radially inhomogeneous extracellular space, and not as much in shape. In the cuff, noisiness only arose at high tortuosity values. In the lower plots, the mean maximum pairwise cross-correlation gives a quantitative confirmation of the higher susceptibility of unmyelinated axons to change their SFAP shape in the presence of tortuosity. Note the different ordinate scales

A more complex axon trajectory caused more complex SFAPs, as it can be seen in Fig. 15. Upper plots of Fig. 15 show superposed SFAP shapes for ten individual axons in both radially inhomogeneous (upper row) and cuff medium (second row).^6^ A summary of SFAP similarity between all ten runs is plotted in the lower row. Unmyelinated SFAPs (Fig. 15a) were especially sensitive to tortuosity. They developed complex, long lasting signals, especially in homogeneous and radially inhomogeneous media. When insulating the nerve, the amplitude of the main SFAP peak became very weak at high tortuosity whilst many small side peaks arose, giving the signal a noisy appearance. Myelinated fibres (Fig. 15b) were more robust to tortuosity – their SFAP shape remained invariant at low and medium *α*-values. Only high degrees of tortuosity could change signal timing and shape; as for unmyelinated axons, the cuff isolated medium let the signal become noisy.

The overall effect of tortuosity to change SFAP shape can be understood by looking at Eq. 10 (same as Eq. 5) and changing it as in Eq. 11 where *s* is the distance along the axon. The longitudinal distance *z*(*s*) along the nerve becomes a function of *s*, shaped by tortuosity. Differences in the potential *ϕ* depending on the radial displacement of the axon were neglected here. The potential profiles of the extracellular media (see Fig. 8) are then both stretched (*z*(*s*) ≤ *s*) and distorted in a degree dependent on tortuosity. Different axons show different susceptibilities to this distortion because of their different active lengths. If the active length is large compared to the spatial frequency of the tortuosity-induced profile distortion, variabilities in *ϕ*(*z*(*s*)) are shadowed. Axons with shorter active length respond to those variabilities making their SFAPs noisier. This explains the difference in susceptibility between axon types.

10 $$\begin{array}{@{}rcl@{}} \phi_{\text{SFAP}} &=& \sum\limits_{z_{i}} \phi(z_{i}) \cdot i \left( t - \frac{z_{i}}{CV}\right) \end{array} $$

11 $$\begin{array}{@{}rcl@{}} \Rightarrow \phi_{\text{SFAP}} &=& \sum\limits_{s_{i}} \phi(z(s_{i})) \cdot i\left( t - \frac{s_{i}}{CV}\right) \end{array} $$

To quantify the influence of *α* on the heterogeneity of SFAP shape, we calculated the pairwise cross-correlation

12 $$ (f \star g)(\tau) = {\int}_{-\infty}^{\infty} f(t) \cdot g(t + \tau) \, dt $$

between normalised SFAP waveforms *s*_*α*,*i*_ from repeated simulation runs whilst keeping *α*, fibre type, and medium unchanged. The mean maximum cross-correlation over all waveform pairs described shape homogeneity:

13 $$ c_{\alpha} = \frac{2}{n\cdot(n-1)} \sum\limits_{k = 0}^{n-1} \sum\limits_{l=k + 1}^{n-1} \max(s_{\alpha, k} \star s_{\alpha, l}).  $$

Figure 15 confirms that a higher *α* caused higher differences in shape (lower *c*_*α*_). As expected from the time course, myelinated SFAPs remained similar even for large *α* whilst unmyelinated ones lost their similarity. Note that this measure does take into account differences in timing or amplitude.

### Stimulation of Tortuous Axons

**Fig. 16:**
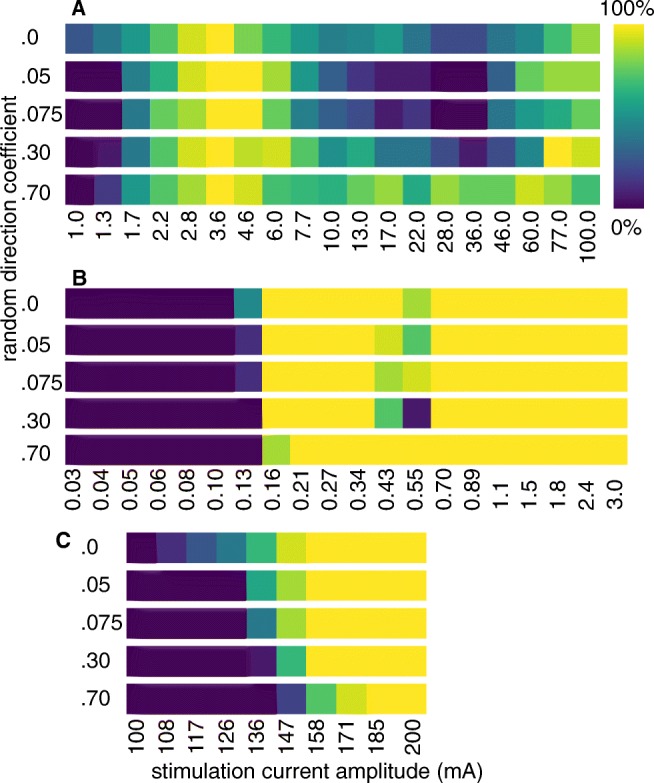
Unmyelinated axons have higher stimulation thresholds and are activated less reliably than myelinated ones. Both bundles consisted of 15 axons with diameter 3 *μ* m and were stimulated with a bipolar electrode of radius 235 *μ* m and pole distance 1 mm using a biphasic pulse of frequency 1 kHz, duration 1 ms and duty cycle 0.5. The extracellular medium was a nerve of diameter 240 *μ* m bathed in oil. **a** Unmyelinated axons started to be activated at 1 mA and showed a peak in activation ratio at about 3 mA. **b** Myelinated fibres had a sharp activation threshold at a much lower current of about 0.15 mA and stayed activated for higher currents. Only when incrementing the stimulation current in very small steps of about 10 nA **c** a slight tortuosity-induced increase in stimulation threshold became visible for them as well

**Fig. 18:**
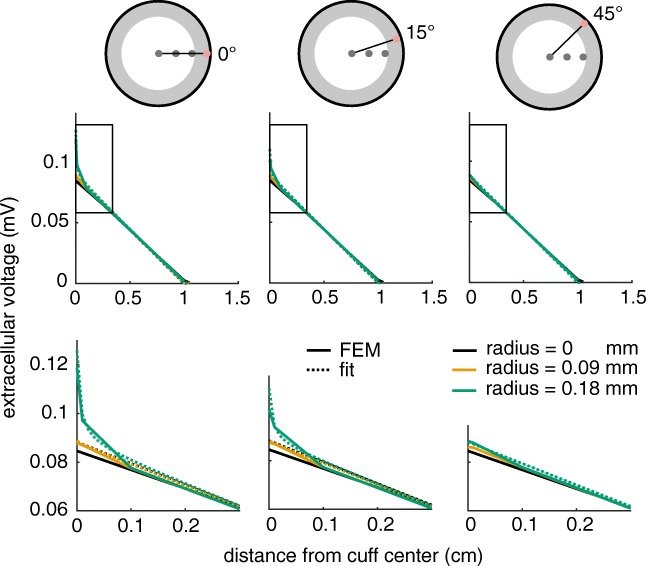
An analytic transmission function implements the relation between current source position and potential for recording in a cuff electrode. In the shown case, a nerve of diameter 480 *μ* m in a cuff of 2 cm length was simulated in the FEM model. Functions are displayed for three different angles between the perpendiculars of source and electrode position onto the bundle guide respectively

Not only the recording from but also the stimulation of axons is influenced by their trajectory. Figure 16 plots the activation ratio of unmyelinated and myelinated fibres for different degrees of tortuosity and different stimulation amplitudes. It shows that firstly, regardless of tortuosity, unmyelinated axons had much higher stimulation thresholds than did myelinated ones. Second, unmyelinated fibres had an optimal stimulation current with a smooth decrease in stimulation efficiency for higher and lower current amplitudes. In the low amplitude range (< 3 mA), perfectly straight axons are activated best. In higher current regimes, very tortuous unmyelinated axons were the most consistently triggered. Stimulation of myelinated axons on the other hand was successful from low amplitudes of about 150 nA and at almost any higher current at all degrees of tortuosity. In Fig. 16c a minor increase in stimulation threshold with tortuosity becomes visible. Therefore, tortuosity affected the activation ratio of unmyelinated axons stronger than it did for myelinated ones.

## Discussion

The open-source simulation framework that we have proposed here for the first time integrates compartmental axon models and numerically solved extracellular space models into a single environment. To make the import of precomputed voltage fields feasible and efficient, the modelled media needed to fulfil certain constraints. One was the geometry that had to be circularly symmetric. Whilst presenting a strong simplification of the extracellular medium, this implementation can be seen as a generic peripheral nerve in which axons can still cluster to fascicles. Another constraint concerned material properties. Displacement currents and therefore frequency dependence of the tissues was not accounted for. Such frequency dependence certainly exists to a certain extent. It can arise from macroscopic structures at constant material properties (dielectric constant *𝜖* and conductivity *σ*) – the epineurium can for instance act as a capacitor. In addition, polarisation at different microscopic levels (Bédard and Destexhe 2009; Martinsen et al. 2002) can render the material properties *𝜖* and *σ* themselves frequency dependent. Such dielectric dispersion is observed in most biological tissues (Gabriel et al. 1996). Ephaptic coupling and neurodiffusive effects were neglected as well.

In terms of axon geometry, we implemented a simple iterative placement mechanism that was fit to microscopy data. To our knowledge this is the first implementation of such automated shape generation for peripheral nerve models. It enabled us to investigate the influence of tortuosity on recordings and stimulation efficiency and indicated that perfectly straight axons are an oversimplification. Our simulation predicted that SFAPs become more complex with increasing tortuosity – an effect that is exploited by spike sorting algorithms which differentiate single units from their SFAP shape. For now, axons were positioned independently from another. As a next step, fibre trajectories could be correlated as observed in microscopy images.

The modular nature of our model allows for an easy comparison of different extracellular media. Building on this functionality, we identified an ideal cuff length for peripheral nerve interfaces. We also showed how the long temporal extent of SFAPs in cuff-insulated media – especially for myelinated axons – makes differentiation of single fibre contributions difficult as overlaps are probable. Overall a cuff therefore increased amplitude but reduced recording precision.

One limitation of the current NEURON simulation is the unmyelinated axon model. Its conduction velocity was too low compared to that reported for mammalian axons. For the overall CAP, the velocity needed to be corrected. Still, the Hodgkin-Huxley parameters are the accepted standard model for unmyelinated axons and more detailed C-fibre models (e.g. Sundt et al. 2015) do not achieve significantly higher conduction velocities either. Parameters of the current model such as membrane capacitance or intracellular resistivity could be adapted to reach the expected conduction velocity but we chose to leave them at their physiological values. If more accurate axon models become available, they can be integrated into PyPNS.

Several steps to improve the model beyond the mentioned limitations are imaginable. First, axons are currently simulated sequentially. For the simulation of closed loop systems interacting with peripheral nerves, the simultaneous simulation of all nerves would be preferable. Second, axon membrane sections only need to be simulated if they are either stimulated or recorded from extracellularly, otherwise the calculation of their highly uniform membrane processes is unnecessary and time consuming. In order to eliminate computational overhead, one could introduce an abstract layer into the simulation in which the position change of spikes along axons is computed based on a known conduction velocity profile. Only for axon segments relevant to stimulation or recording, would the full membrane process be simulated.

In conclusion, a unified computer model of a generic peripheral nerve was developed. It combined an efficient calculation of extracellular potentials in inhomogeneous media from precomputed potential fields with compartmental axon models in a convenient Python module. The model was validated against experimental data and used to investigate the effects of conductivity inhomogeneities on amplitude and frequency content as well as the influence of axon tortuosity on both recording and stimulation. We hope that the simulation framework presented here, PyPNS, becomes a useful tool for researchers working on peripheral nerves, nerve stimulation, and its medical applications, and envision that the toolbox could be augmented by multiple branches, organ models, and a variety of specific axon models matched to fibre types found in different parts of the peripheral nervous system, to facilitate this.

## Information Sharing Statement

The latest version of our toolbox PyPNS (RRID:SCR_016336) can be accessed over GitHub: http://github.com/chlubba/PyPNS. The version this paper is based on is stored on Zenodo: 10.5281/zenodo.1204836. Scripts for the figures are as well maintained on GitHub: http://github.com/chlubba/PyPNS-PaperFigureshttp://github.com/chlubba/PyPNS-PaperFigures.

## Figures and Tables

**Fig. 17:**
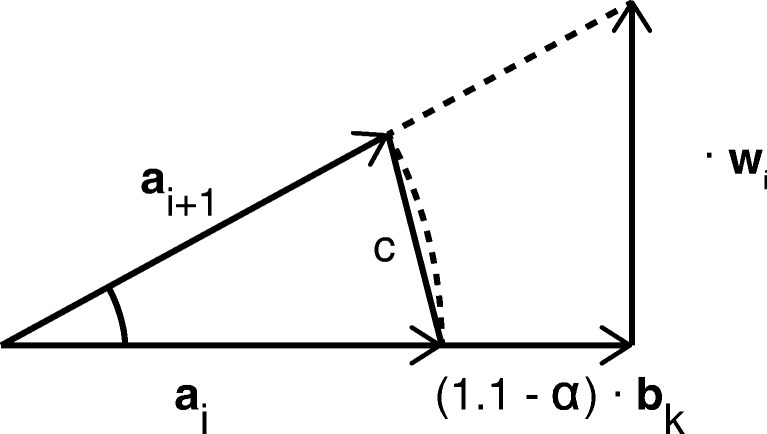
If bundle **b**_*k*_ and current axon segment **a**_*i*_ have a fixed relation, e.g. parallel, the expected distribution of segment direction differences *c* = ||**a**_*i*_ −**a**_*i*+ 1_|| can be easily obtained from the distribution of ||**w**|| ($\mathcal {P}$) by their geometrical relation

## Appendix A: Calculation of the Random Component of the Axon Placing Algorithm

The random vector **w**_*i*_ in Eq. 3 is split into an inward pointing radial **w**_rad_ and a tangential component **w**_tan_ (14), both weighted independently with a weight drawn from a distribution $\mathcal {P}$ (15, 16). $\mathcal {P}$ can be either a uniform distribution $\mathcal {U}(-1, 1)$ between − 1 and 1 or a normal distribution $\mathcal {N}(\mu , \sigma )$ with *μ* = 0 and *σ* = 0.33 (sigma chosen to have 99.7% of all values in the range [− 1, 1]). When the radial distance between axon segment and bundle guide *d* approaches the bundle radius *r*_**bundle**_, the radial component **w**_rad_ becomes more inward directed (16) and thereby ensures that the axon stays inside the nerve. One linear implementation of the correction factor *e* is shown in Eq. 17. The parameter *r*_corr_ defined the relative radius from which on the correction should begin, set to 0.7 in our simulation; *e*_max_, set to 2 by default in PyPNS, limits the correction.

14 $$\begin{array}{@{}rcl@{}} && \mathbf{w}_{i} = \frac{\beta_{\text{rad}} \cdot\mathbf{w}_{\text{rad}} + \beta_{tan}\cdot\mathbf{w}_{\tan}}{||\beta_{\text{rad}}\cdot\mathbf{w}_{\text{rad}} + \beta_{\tan}\cdot\mathbf{w}_{\tan}||} \end{array} $$

15 $$\begin{array}{@{}rcl@{}} && \beta_{tan} \sim \mathcal{P} \end{array} $$

16 $$\begin{array}{@{}rcl@{}} && \beta_{rad} \sim \mathcal{P} - e \end{array} $$

17 $$\begin{array}{@{}rcl@{}} && e = \min(1, \max(0, \frac{d/r_{\text{bundle}} - r_{\text{corr}}}{1-r_{\text{corr}}})) \cdot e_{\max} \end{array} $$

## Appendix B: Generation of Simulated *c*-Distributions

To directly translate ||**w**||-distributions ($\mathcal {P}$) to distributions of the normed difference in direction of consecutive axon segments *c* = ||**a**_*i*_ −**a**_*i*+ 1_|| projected onto a 2D-plane, we made the simplifying assumption that **b**_*k*_ and **a**_*i*_ are aligned. By doing so || **a**_*i*_ + (1.1 − *α*) ⋅**b**_*k*_||) (see Eq. 3) becomes (2.1 − *α*) ⋅||**a**_*i*_||. Following Fig. 17, it is easily shown that then ||**w**|| relates to *c* as

18 $$ c = 2 \cdot ||\mathbf{a}|| \cdot \sin\left( \frac{1}{2}\cdot \arctan \left( \frac{||\mathbf{w}||}{||\mathbf{a}||} \frac{\alpha}{2.1 - \alpha} \right)\right).  $$

## Appendix C: Fitted Cuff Transmission Function

For extracellular recording in a cuff, a transfer function between current point source position and the potential at an electrode longitudinally centrally placed in the cuff was fit. Input variables describe the spatial relation between source and receiver position. As apparent from Fig. 18, the relation is strongly linear with an additional peak for low distances between current source and potential receiver – facilitating the fit of a transfer function.

The static potential was therefore described as a linear component *f*_lin_(*z*) plus a non-linear peak *f*_peak_(*z*). Equations 21–23 implement this characteristic for *ϕ* in mV with variables *r*_axe_ radial axon displacement in m, *α* angle between axon displacement direction and electrode perpendicular on the nerve centre in rad and *z* longitudinal distance between electrode and axon in m. The transfer function is parametrised with *r*_1_ for the inner radius of the nerve in m, *a* and *b* for maximum peak amplitude and steepness, *c* for maximum of triangular component and *d* half the cuff length in m. The left and right borders of the *f*_lin_(*z*)-function were smoothed with a moving average of width *c*/20.

19 $$\begin{array}{@{}rcl@{}} f_{\text{lin}}(z) \!&\!=&\! \max(0, c\cdot(1-|z/d|)) \end{array} $$

20 $$\begin{array}{@{}rcl@{}} f_{\text{peak}}(z) \!&\,=\,&\! \frac{a}{|z| \,+\, b} \end{array} $$

21 $$\begin{array}{@{}rcl@{}} f_{\text{peak},r}(r_{\text{axe}}) \!&\,=\,&\! \min(1, (r_{\text{axe}} / r_{1})^{5}) \end{array} $$

22 $$\begin{array}{@{}rcl@{}} f_{\text{peak},\alpha}(\alpha) \!&\!\,=\,&\! \max(0, \!(1\!\,-\, |\!\bmod\!(\alpha\,+\,\pi, 2\pi) \!\,-\, \pi|)/\pi \!\!\cdot\! 5) \end{array} $$

23 $$\begin{array}{@{}rcl@{}} \phi(z, \alpha, r_{\text{axe}}) \!&=&\! tr(z) + p(z) \cdot pf_{r}(r_{\text{axe}}) \cdot pf_{\alpha}(\alpha) \end{array} $$

**Table 4:** Parameters of the fitted transmission function for cuff recordings

Parameter	Value
*r*_1_	1.9 ⋅ 10^− 4^
*a*	2.5 ⋅ 10^− 9^
*b*	5 ⋅ 10^− 5^
*c*	8.83 ⋅ 10^− 4^
*d*	0.01

Spatial input variables in m, angle in rad, output in mV

## Footnotes

1 See information sharing statement at the end for accessing the toolbox.

2 Point sources were given preference over the line source approximation to enable our efficient precomputation of extracellular potentials.

3 A homogeneous but anisotropic medium can in fact be modelled analytically using a conductivity tensor (Nicholson and Freeman 1975; Goto et al. 2010). A combination of inhomogeneities and anisotropy is not feasible, however.

4 For a nerve of radius 200 *μ* m, a longitudinal length coverage of 20.000 *μ* m and a source position grid step of 20 *μ* m this would mean approximately 300,000 simulations, each taking at least 30 min on a single core of a state of the art workstation, totaling to a computation time of over 17 years. The result would occupy 12 TB of RAM.

5 Electrode radius was chosen to be slightly smaller than nerve radius to maintain a small distance to the non-conducting insulation layer surrounding the nerve.

6 The homogeneous medium produced very similar SFAP shapes in time as the radially inhomogeneous medium. Therefore the traces are not shown. The similarity summary in the lower row was plotted for all three media.
